# Antimicrobial Resistance in Typhoidal *Salmonella*: Around the World in 3 Days

**DOI:** 10.1093/cid/ciaa366

**Published:** 2020-07-29

**Authors:** Senjuti Saha, Mohammad Saiful Islam Sajib, Denise Garrett, Farah N Qamar

**Affiliations:** 1 Child Health Research Foundation, Department of Microbiology, Dhaka, Bangladesh; 2 Department of International Health, Johns Hopkins Bloomberg School of Public Health, Baltimore, Maryland, USA; 3 Sabin Vaccine Institute, Washington, USA; 4 Department of Pediatrics and Child Health, Karachi, Pakistan

**Keywords:** typhoid, antimicrobial resistance, typhoidal *Salmonella*, Typhi, Paratyphi

## Abstract

With the increasing antibacterial resistance in typhoidal *Salmonella* and the dearth of novel antimicrobials on the horizon, we risk losing our primary defense against widespread morbidity and mortality from enteric fever. During 26–28 March 2019, researchers from around the world came together in Hanoi, Vietnam, and shared some of their latest findings on antimicrobial resistance. From the 258 abstracts presented at the conference, at least 50 discussed phenotypic and genotypic characteristics of antimicrobial resistance in typhoidal *Salmonella*, covering data of at least 24 different countries, spanning 5 continents. Here, we summarize the key findings, focusing on our global journey ahead.

## HUMANS VS TYPHOIDAL SALMONELLA: THE NEVER-ENDING ARMS RACE

The chronicle of war between the human population and typhoidal *Salmonella* (*Salmonella enterica* serovars Typhi [*S.* Typhi] and Paratyphi [*S.* Paratyphi] A, B, and C) goes back centuries [1–3]. In the 1800s and the early 1900s, with mortality rates of about 30%, enteric fever (typhoid and paratyphoid) ravaged many parts of the world, including elite neighborhoods in cities like New York in North America and Oxford in the United Kingdom [3, 4]. The ferocity of this ghastly disease dwindled radically with improved water, sanitation, and hygiene (WASH) in resource-rich countries and with the advent of antibiotics, which decreased mortality to < 1% [3, 5]. Today, the overwhelming burden of enteric fever rests disproportionately on low- and middle-income countries (LMICs), specifically in South Asia and sub-Saharan Africa [6]. The WASH improvements seen in the developed countries have not been effectively implemented in LMICs; hence, the primary weapon to fight the disease is antibiotics. However, with increasing use of antibiotics in these countries, antimicrobial resistance (AMR) is steadily rising, and individual resistance to every widely used antibiotic to treat enteric fever has been reported. With increasing AMR and the slow pace of WASH improvements in LMICs, there is rising fear of untreatable infections and returning to the high mortality rates experienced in the preantibiotic era.

The first antibiotic to treat typhoid, chloramphenicol, was introduced in 1948 [7]. As resistance to chloramphenicol started emerging, ampicillin and co-trimoxazole were introduced in the treatment protocol in the 1970s [8]. However, by the 1980s, reports of *S.* Typhi resistant to all 3 drugs—ampicillin, chloramphenicol, and co-trimoxazole (also known as the first line of antibiotics)—started emerging. The simultaneous resistance to these 3 drugs is defined as multidrug resistance (MDR), and this was linked to the emergence of the H58 haplotype (or genotype 4.3.1) that carried an IncHI1 plasmid with multiple antibiotic resistance genes [9–11]. As a result, the primary treatment for enteric fever shifted to fluoroquinolones, but not surprisingly, with widespread use, soon there were reports of decreasing fluoroquinolone susceptibility due to rise of point mutations in the gyrase and topoisomerase genes [12, 13]. This left third-generation cephalosporins and azithromycin as 2 of the very few reliable options to treat enteric fever.

The first cephalosporin-resistant cases were reported from Bangladesh in 1998 and 2001, but they appeared to be isolated events [14, 15]. In 2016, Pakistan was hit by a typhoid outbreak caused by an extensively drug-resistant (XDR) strain of *S.* Typhi that was resistant to chloramphenicol, ampicillin, co-trimoxazole, streptomycin, fluoroquinolones, and third-generation cephalosporins [16]. This XDR strain encodes a chromosomally located resistance region and carries a plasmid encoding additional resistance elements, including the *bla*_CTX-M-15_ extended-spectrum β-lactamase, and the *qnrS* fluoroquinolone resistance gene. The primary oral drug available to treat XDR *S.* Typhi in the outpatient department, where the majority of the patients are treated, is azithromycin [17]. Meropenem, an injectable antibiotic, is used in the inpatient department to treat hospitalized children.

In January 2018, the World Health Organization adopted a recommendation for use of typhoid conjugate vaccines (TCVs) in settings with high burden of typhoid and has prequalified the first TCV [18, 19]. A recent phase 3 trial in Nepal illustrated that a single dose of TCV was immunogenic and effective in reducing bacteremia by *S.* Typhi in children 9 months to 16 years of age [20]. Countries are now facing important decisions about whether to provide TCVs and at what geographic scale, and in the light of the recent outbreak in Pakistan, these decisions are of great consequence for fighting the further rise and spread of AMR.

The 11th International Conference on Typhoid and Other Invasive Salmonelloses, held in Hanoi, Vietnam, from 26 to 28 March 2019, brought together researchers from around the world to present their most recent findings, take part in discussions, and speculate on the way forward as a global community. On the backdrop of the ongoing XDR outbreak in Pakistan, not surprisingly, a large proportion of the presented work was dedicated to the growing crisis of AMR in typhoidal *Salmonella*. From 258 abstracts, at least 50 abstracts pertained to genomic and phenotypic characteristics of AMR. These 50 abstracts covered data from at least 24 different countries, spanning 5 continents. The data presented in this conference are some of the most globally represented and up-to-date.

## *SALMONELLA ENTERICA* SEROVAR TYPHI: THE PROTAGONIST

At least 25 presentations focused on different aspects of AMR pertaining to *S.* Typhi, with 6 presentations dedicated to the ongoing XDR typhoid outbreak in Pakistan. The outbreak, detected in 2016, has affected > 10 000 people in Pakistan [21–23]. Farah Qamar and her team recently published that risk factors for acquiring XDR S. *Typhi* include being male, eating outside the home, exposure to a patient with *S.* Typhi infection, and a history of antimicrobial use [24]. The XDR cases clustered around sewage lines, and *S.* Typhi DNA was detected in about a fourth of community water sources, reasserting the importance of access to clean water for decreasing the burden of enteric fever.

Researchers, clinicians, and public health officials in Pakistan have come a long way since the group last met 2 years ago in Kampala, during the 10th conference in 2017. The recently approved TCV has already been introduced in the affected regions, where thousands of children are currently being vaccinated. Following the conference, on 15 November 2019, Pakistan became the first country to introduce TCV in its national immunization program to control the outbreak and further spread of AMR [25]. Mudasser Hussain’s team from Pakistan drew attention to the importance of decreasing injudicious use of antibiotics to prevent future outbreaks of this severity and scale.

**Figure 1.**
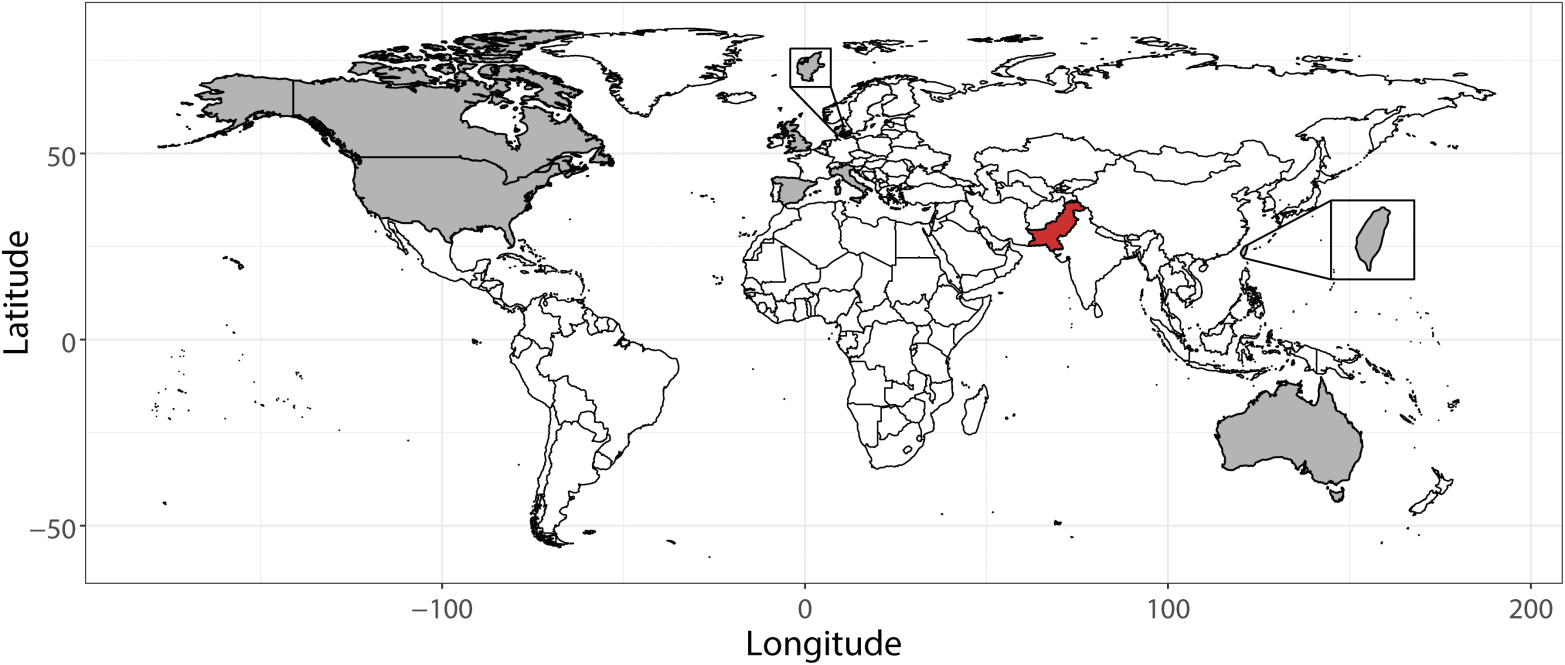
Global spread of extensively drug-resistant (XDR) *Salmonella* Typhi strains that originated in Pakistan. In red is Pakistan, and in gray are countries where travel-related XDR typhoid cases have been identified up to 20 March 2020. We conducted a PubMed search using the terms “*Salmonella* Typhi”; “XDR”; “typhoid”; “outbreak”; and “travel” in different combinations. We also used the Google search engine to find additional literature or other reliable sources that contained reports on extensively drug-resistant *Salmonella* Typhi. A total of 7 PubMed indexed research and 1 news article (Taiwan XDR case) have been incorporated in this map [16, 26–31].

Since the report of the XDR typhoid outbreak, there have been warranted fears of global spread of the XDR strain. At the conference, Grace Appiah of the United States (US) Centers for Disease Control and Prevention presented 5 travel-related XDR cases in the US. As the outbreak continues in Pakistan, additional cases of XDR *S.* Typhi have also been reported from the United Kingdom, the US, Australia, Denmark, Canada, Spain, Taiwan, and Italy [16, 26–31] (Figure 1).

Apart from Pakistan, AMR profiles of *S.* Typhi were presented from at least 23 countries. Of specific importance are the multicountry Typhoid Fever Surveillance in Africa Program (TSAP) and the Surveillance of Enteric Fever in Asia Project (SEAP) studies [32, 33]. TSAP is conducted in 10 different countries (Burkina Faso, Ghana, Ethiopia, Kenya, Guinea-Bissau, Madagascar, Senegal, South Africa, Tanzania, and Sudan), and Se Eun Park of the International Vaccine Institute presented that 52% of typhoidal *Salmonella* strains circulating in the region are MDR. Similarly, Susan Kavai of the Kenya Medical Research Institute presented that 56% of the Typhi strains isolated in Kenya are MDR and 18% are fluoroquinolone resistant. However, these rates appear to vary between countries in Africa. Peter Adikwu’s team in Nigeria detected 27% MDR and 56% fluoroquinolone-nonsusceptible *S.* Typhi strains. Tapfumanei Mashe from the Ministry of Health and Child Care Zimbabwe described a gradual increase in fluoroquinolone resistance (from 0% in 2012 to 22% in 2017), and pointed toward a potential outbreak where all the strains were fluoroquinolone resistant.

SEAP is conducted in 3 countries in South Asia (Bangladesh, Pakistan, and Nepal). Preliminary AMR data presented by Muhammad Tahir Yousafzai of this multicountry study depicted low rates of MDR (18% in *S.* Typhi and 1% *S.* Paratyphi A) but high rates of fluoroquinolone nonsusceptibility in both Bangladesh and Nepal (> 90%). In Pakistan, on the other hand, 85% of the circulating *S.* Typhi strains were found to be XDR. Interestingly, several independent studies from India and Myanmar (Sriparna Samajpati et al, Bhaskar Shenoy et al, and Tin Ohn Myat et al) noted a reduction of MDR and high prevalence of fluroquinolone nonsusceptibility among *S.* Typhi strains in these countries.

With the rise in fluroquinolone resistance and increasing cases of XDR *S.* Typhi, azithromycin is the only reliable oral drug available against typhoid. Historical reports of azithromycin resistance are rare. However, its increasing use places selective pressure for the emergence and spread of azithromycin-resistant isolates. Researchers of the Strategic Typhoid Alliance Across Africa and Asia (STRATAA) study reported azithromycin resistance of 21% in Bangladesh and 2.8% in Nepal, but nonexistent in Malawi. Senjuti Saha’s team from the Child Health Research Foundation of Bangladesh dedicated a talk to azithromycin resistance and described 12 cases of azithromycin-resistant *S.* Typhi strains and the molecular mechanism conferring such resistance [34]. Her team showed that azithromycin resistance is gradually increasing in Bangladesh, and the molecular basis of this resistance is a single point mutation in the AcrB protein, an efflux pump, at position 717. This is the first report demonstrating the impact of this mutation in conferring azithromycin resistance in a clinical setting. All azithromycin-resistant strains were nonsusceptible to ciprofloxacin, ampicillin, co-trimoxazole, and chloramphenicol, and the only oral drug available for treatment was third-generation cephalosporin. As azithromycin is the only oral drug available to treat XDR *S.* Typhi, acquisition of the plasmid that confers cephalosporin-resistance in XDR strains by a Bangladeshi azithromycin-resistant strain or rise of a point mutation in the XDR strains can bring us to the brink of losing all antimicrobial weapons against typhoid. This poses serious threats to the health system of LMICs*—*an azithromycin-resistant XDR strain would shift enteric fever treatment from the outpatient department, where patients are currently treated with oral azithromycin, to inpatient departments to be treated with injectable antibiotics such as carbapenems, weighing down already struggling health systems [35].

## *SALMONELLA ENTERICA* SEROVAR PARATYPHI A: THE SIDEKICK

Typhoid and paratyphoid fever are often considered a single disease, with the management of paratyphoid fever based on lessons learned from typhoid studies. However, there is growing realization in the scientific community that they are not identical diseases [36]. For example, *S.* Paratyphi A (the primary cause of paratyphoid fever) has different AMR profiles than that of *S.* Typhi. A previous study from Bangladesh showed that MDR in Paratyphi A strains appears to be much less prevalent than in *S.* Typhi [37]. Furthermore, in comparison to children with typhoid, children with paratyphoid are more likely to be older, and are more often treated in the outpatient department [36]. As TCV does not protect against paratyphoid fever, and because *S.* Typhi and *S.* Paratyphi A exhibit different AMR profiles, disease dynamics and effectiveness of empirical therapy are likely to change in the near future. Therefore, gaining further knowledge about *S.* Paratyphi A is crucial, and in this conference, at least 23 presentations focused on AMR trends/patterns of *S.* Paratyphi A in addition to *S.* Typhi, and 1 presentation was dedicated to Paratyphi A only.

Corroborating with the published literature, the majority of studies noted that MDR (also defined as resistance to ampicillin, chloramphenicol, and co-trimoxazole) is uncommon in *S.* Paratyphi A strains. Presentations from Bangladesh, India, and Myanmar provided evidence of complete absence of MDR *S.* Paratyphi A in these 3 countries. However, Asif Khaliq from Aga Khan University Hospital in Pakistan reported the MDR rate of *S.* Paratyphi A at 53%. Such high rates have not been reported elsewhere, including in the SEAP study, where the current MDR rate of Paratyphi A is reported at 1% in Pakistan, and hence requires further investigation.

Saiful Islam Sajib presented data of about 2000 *S.* Paratyphi A strains from Bangladesh. This is the largest known historical dataset for Paratyphi A, and with data from the period 1999–2016, he described absence of MDR but a rising trend in the minimum inhibitory concentration of ciprofloxacin among the Bangladeshi isolates. He demonstrated that low-cost restriction fragment length polymorphism methods can be used to detect and monitor the circulating gyrase A mutations that lead to fluroquinolone nonsusceptibility in *S.* Paratyphi A. Like *S.* Typhi, almost all discussions concluded that decreased susceptibility to fluoroquinolones is also commonly exhibited by *S.* Paratyphi A, and hence ciprofloxacin should not be a treatment of choice.

A study by Marie Chattaway from Public Health England reported a travel-related ceftriaxone-resistant paratyphoid fever case, apparently originating in Bangladesh. This is the first report of ceftriaxone-resistant Paratyphi A; however, none of the SEAP, STRATAA, or other long-term, extensive prospective studies conducted in Bangladesh, some for decades, reported any such observation. The molecular basis of this resistance was the carriage of a plasmid containing the *bla*_CTX-M-15_ extended-spectrum β-lactamase gene, similar to the one detected by Djeghout et al in ceftriaxone-resistant *S.* Typhi [15]. If the findings of this study are indeed correct, the phenomenon is of serious concern and should be investigated further.

Similar to *S.* Typhi, azithromycin resistance is also emerging in *S.* Paratyphi A, and this was reported by authors from Bangladesh, India, and Nepal. However, these results should be cautiously examined. Dabet Rynga from India noted that assessing azithromycin resistance in *S.* Paratyphi A is difficult, as guidelines for azithromycin disk diffusion and minimum inhibitory concentration interpretive criteria by the Clinical and Laboratory Standards Institute exist only for *S.* Typhi, and not for *S.* Paratyphi A. The mechanism of azithromycin resistance in *S.* Paratyphi A is conferred by the same single point mutation in the AcrB efflux pump as that in *S.* Typhi (described above) [34].

## THE PATH FORWARD

*Salmonella* Typhi is estimated to cause 10.92 million illnesses and 105 500 deaths, and *S.* Paratyphi A to cause 3.39 million illnesses and 19 100 deaths every year [6]. Two-thirds of all etiologies of bloodstream infections from cases > 2 months of age in endemic countries comprise of *S.* Typhi or *S.* Paratyphi A [38]. With increasing antibacterial resistance in typhoidal *Salmonella* and the dearth of novel antimicrobials on the horizon, we risk losing our primary defense against enteric fever, and returning to the preantibiotic era when the mortality rate was about 30%.

Introduction of the new TCV in endemic countries will be key in controlling typhoid. Data from the phase 3 clinical trial in Nepal are promising, and Pakistan has recently added TCV to its national immunization program. However, considering the overwhelming burden of typhoid, control of typhoid, and spread of AMR will require multipronged approaches. First, at present, typhoid vaccine is manufactured by only 1 company, and it may prove difficult for them to supply the millions of doses that will be required by all endemic countries. Therefore, deliberate strategies by companies, donors, and policymakers are very important for sustainable production and supply of TCV. Second, in addition to vaccines, to interrupt the trend of increasing AMR, local antibiotic stewardship led by public health officials and clinicians will be crucial. As AMR patterns vary considerably between countries, empirical treatment guidelines should be designed based on local data. Third, we must also not lose sight of the fact that paratyphoid also poses a high burden, and rising AMR of *S.* Paratyphi A is also of serious concern. Last but not least, we must remember that, historically, WASH interventions have been the most successful in controlling enteric fever. Hence, it is imperative for local policy makers and international donors to continue their efforts and investments on WASH, which will help in the control of both typhoid and paratyphoid.

The fight against enteric fever calls for collective and comprehensive global and local action. Partnerships between local and international clinicians, public health officials, scientists, policy makers, and donors are imperative to win the arms race against rising AMR in enteric fever.

## Notes

***Financial support.*** This work was supported by the Bill & Melinda Gates Foundation (grant number OPP1182032).

***Supplement sponsorship.*** This supplement is funded with support from the Coalition against Typhoid Secretariat, housed at the Sabin Vaccine Institute in Washington, DC and made possible by a grant from the Bill & Melinda Gates Foundation.

***Potential conflicts of interest.*** The authors: No reported conflicts of interest. All authors have submitted the ICMJE Form for Disclosure of Potential Conflicts of Interest.
